# A New Ready-to-Eat Product Based on Enzymatically Peeled ‘Hernandina’ Clementine Segments and Citrus Syrup

**DOI:** 10.3390/foods12213977

**Published:** 2023-10-30

**Authors:** Huertas M. Díaz-Mula, Juan P. López, María Serrano, María T. Pretel

**Affiliations:** 1 Department of Biología Aplicada, Escuela Politécnica Superior de Orihuela (EPSO), Universidad Miguel Hernández, Carretera Beniel-Orihuela, Km 3.2, 03312 Orihuela, Alicante, Spain; h.diaz@umh.es (H.M.D.-M.); juanpedrolopez_10@hotmail.com (J.P.L.); m.serrano@umh.es (M.S.); 2 Instituto Universitario de Investigación e Innovación Agroalimentaria y Agroambiental (CIAGRO), Universidad Miguel Hernández, Carretera Beniel-Orihuela, Km 3.2, 03312 Orihuela, Alicante, Spain

**Keywords:** enzymatic peeling, citrus segments, sensory properties, vitamin C, pasteurization

## Abstract

Ready-to-eat fresh fruit have an increasing presence in international markets due to their convenience and health benefits. However, these products are highly perishable and efficient technologies to increase their shelf life are needed. In the present research, different citrus fruit species and cultivars from organic farming were assessed to obtain enzymatically peeled citrus segments. The best results in terms of segment quality were observed for ‘Hernandina’ clementine, which was chosen to make a new ready-to-eat product based on peeled citrus segments that were packaged in glass jars with a light syrup made of citrus juice and organic sugar cane. Different citrus juice mixtures were assayed and the most appreciated syrup, based on the sensory scores given by panellists, was that containing 50–50 (*v*/*v*) of ‘Fino’ lemon and ‘Hernandina’ clementine juices. In addition, different pasteurization treatments were assessed for their effects on conserving the safety, nutritional quality and sensory properties of the product during cold storage. The results show that pasteurization treatment at 50 °C for 45 min was sufficient to prevent microbial contamination with mesophilic and psychrophilic aerobic bacteria or yeast and mould and to maintain sensory properties until five weeks of storage at 4 °C. In addition, only a 10% reduction in vitamin C concentrations was observed in fresh-segments or syrup until the end of the storage period, showing that a high bioactive compound content and health benefits were conserved in the new ready-to-eat product after pasteurization and prolonged cold storage.

## 1. Introduction

Diets rich in fruit and vegetables have been proved to have health benefits by reducing the risk of suffering from degenerative diseases [1,2]. In particular, the health beneficial effects of citrus fruits have been attributed mainly to ascorbic acid and flavonoids, such as naringenin and naringin [3,4]. In recent times, changes in lifestyles have led to increased consumer demand for ready-to-eat fruit products to satisfy their requirements for balanced and healthy diets [5]. As a result, minimally processed fresh fruits, which are washed, peeled, cut, packaged and ready to eat, have an increasing presence in international markets as they provide multiple advantages for the consumers, such as saving time or being easy to use [6]. In addition, they can be used to encourage children and adolescents to increase the fruit and vegetable content in their diets since they are attractive, already peeled and cut and easy to eat [7]. However, these products are highly perishable with a short shelf life, even under cold storage. Thus, more research is needed to satisfy the consumers’ expectations for fresh, low-processed fruits with high phytonutrient content, but also with quality and safety properties that are preserved as long as possible. Therefore, different technologies have been used to retard their perishability, such as heat treatments, modified atmosphere packaging (MAP), edible coatings, high pressure, gamma or ultraviolet radiation or electrolyzed water, among others [6,8,9]. Other kinds of ready-to-eat vegetable products are preserved by a mild heat treatment (pasteurization) after washing, slicing, chopping or shredding into 100% usable product. These products are adapted to new consumer needs, since they are kept refrigerated and are directly ready for consumption [10]. On the other hand, consumption of organic fruit has been continuously increasing in recent years, due to social concern regarding environmental and economic problems presented by the intense production activity of the agro-industrial sector [11]. 

Conventional industrial processes used for citrus peeling consist of the manual or mechanical separation of the rind and the subsequent chemical degradation of the remaining albedo and segment membranes. To deal with this process, enzymatic peeling utilizes an enzymatic preparation containing natural cell wall hydrolases. Enzymatic peeling has certain advantages compared with conventional methods, such as improving the yield and quality of the fruit segments obtained, maintaining the fruit’s original flavour and texture, and reducing water consumption and contaminants, making this method a more sustainable and eco-friendlier alternative than the traditional process [12,13,14,15]. For instance, segments from ‘Cadenera’, a traditional orange cultivar, were obtained by enzymatic peeling and packaged in micro-perforated films (with nonselective permeability) made of polypropylene, and segment quality and microbiological safety were maintained for 7 days of storage at 4 °C [12]. Similar results were reported by Barrios et al. [16] in enzymatically peeled ‘Valencia’ orange segments packaged in non-perforated polypropylene bags. However, the storage of peeled citrus segments in these modified atmosphere packages has some limitations, namely, the use of plastic films with the associated problems of environmental pollution and the short shelf life of the ready-to-eat product. Thus, new technologies with potential application in the agro-food industry are needed to preserve these products for longer periods while avoiding environmental concerns. Such technologies could include the use of thermal treatments to increase the microbiological safety and storage in glass jars as eco-friendlier packaging.

Consumers usually judge the quality of ready-to-eat products based on their appearance and freshness at the time of purchase. Therefore, the heat treatments performed on these products to guarantee food safety must satisfy the consumers’ requirements in terms of fruit texture and flavour [8,17,18]. The most common treatment to control pathogen growth in ready-to-eat fruit and vegetable products is pasteurization, applied alone or together with other techniques, such as modified atmosphere, refrigeration or other innovative technologies [8,19]. Pasteurization operates at temperatures below 100 °C, usually ranging from 40 to 60 °C, by treatments with hot air or immersion in hot water for different periods depending on the volume of the fruit product, leading to increased useful life while minimally altering the product quality [20,21]. However, although these treatments are useful to ensure food safety, the application of heat to fresh products under inadequate conditions, can cause serious quality deterioration such as colour and texture degradation, or vitamin loss [8,22]. Thus, appropriate selection of the optimal heat treatment for ready-to-eat fruit is needed in order to maintain its best sensory properties and general quality, in addition to its microbiological safety. 

Thus, the main goal of this research was to obtain a ready-to-eat new product composed of enzymatically peeled organic citrus fruit and a light syrup and packaged in glass jars. Firstly, five citrus fruit species and cultivars were assayed to obtain enzymatically peeled citrus fruit segments with good sensory properties. Then, different syrups were made with nine mixtures of lemon and clementine juices and tested for their sensory properties. Finally, the new ready-to-eat product, based on peeled citrus segments packaged in glass jars with a suitable light syrup, was submitted to different pasteurization treatments to determine which treatment is most effective at preserving the product’s microbiological safety, and nutritional and sensory quality properties during storage at cold temperature. 

## 2. Materials and Methods

### 2.1. Plant Material

Citrus fruits including ‘Navel’ sweet orange (*Citrus sinensis* (L.) Osbeck); ‘Hernadina’ clementine (*Citrus* × *clementine*); ‘Orogrande’ clementine (*Citrus* × *clementine*); ‘Star Ruby’ grapefruit (*Citrus paradisi* Mcfad) and ‘Fino’ lemon (*Citrus limon* (L.) Osbeck) were harvested from a commercial field owned by the ECO-CITRIC company (Orihuela, Alicante, Spain). All the citrus species and cultivars were in close plots under similar environmental and agronomic conditions and grown under an organic farming production system.

### 2.2. Enzymatic Peeling of Citrus Cultivars

Thirty fruits from each citrus cultivar, harvested at commercial ripening stage and without external damage, were selected. The fruits were rolled over a 1 m^2^ wood plate with cylindrical metal spikes (5 mm long, 1 mm in diameter and with 10 mm of separation) in order to homogeneously perforate the fruit surface [13,23]. Then, fruits in lots of 10 were immersed in a water bath at 40 °C for 30 min to ensure that in internal fruit tissues reached this temperature. Thereafter, the fruit were transferred to a vacuum tank containing 9 L of 1 g L^−1^ Peelzym II solution (Novo Nordisk Fermemt Ltd.; Dittingen, Switzerland), a commercial pectolytic product with pectinase and polygalacturonase activities produced by *Aspergillus niger*, at 40 °C and three vacuum pulses of two min at 57 kPa were applied. The fruit were left in the peeling solution for 10, 20 or 30 min incubation times at atmospheric pressure and 40 °C. The peeling efficiency after the different incubation times was independently evaluated by five judges (each judge evaluated two fruit) by assessing the following parameters according to Pretel et al. [13]: percentage of albedo not attacked by the enzymatic solution; easiness of skin removing (albedo plus flavedo), from very easy (5) to very difficult (1); easiness of segment separation, from very easy (5) to very difficult (1); segment firmness, from high (5) to low (1); percentage of viable segments (segments without defects); and global acceptance (percentage). The experiment was replicated three times. The citrus cultivar (‘Hernandina’ clementine) and an incubation time of 10 min achieved the highest quality segments in this experiment. This cultivar and incubation time were selected to produce a new ready-to-eat, minimally processed food product based on citrus segments in light sugar syrup. The product’s organoleptic and microbial quality traits were evaluated during storage at 4 °C.

### 2.3. Sugar Syrups

Nine different syrups were made with ‘Hernandina’ clementine (HCJ) and ‘Fino’ lemon juices (FLJ) in different proportions, from 10 to 90%. The juices were obtained with a manual juicer from fruit harvested at commercial ripening stage from the plot grown under organic farming mentioned in Section 2.1. The initial °Brix levels of the juice mixtures were measured and then cane sugar from organic farming, purchased from Rincón del Segura S.L. company (Elche de la Sierra, Albacete, Spain), was added to obtain a light syrup with 14 °Brix, in order to comply with the light syrup specifications regulated by Real Decreto 2420/1978 [24]. These syrups were evaluated by a sensory panel comprising 10 semi-trained non-professional panellists (5 female and 5 males, aged 25–50), who were trained before the experiments in order to achieve a suitable level of knowledge of the details of the sensory analysis, according to the standard ISO 8586:2012 (This standard has been revised by ISO 8586:2023). The sensory evaluation was performed in a tasting laboratory with an individual cabin provided for each panellist, under fluorescent lighting and at a room temperature of 22  ±  2 °C. The judges assessed the following quality traits by giving scores from 0 (do not like at all) to 10 (like very much): colour, odour, sweetness, aroma, overall impression and purchase intention. Scores for sourness ranged from 0 (no sourness) to 10 (extremely sour). The panellists were asked to drink mineral water to refresh their senses between samples. The experiment was replicated three times.

### 2.4. Elaboration of the Ready-to-Eat ‘Henandina’ Clementine Segments in Syrup

Glass jars of 250 mL were filled with 150 g of enzymatically peeled ‘Hernandina’ clementine segments (obtained as indicated above) and 80 mL of syrup composed of FLJ and HCJ (50:50 *v*:*v*, selected according to the results of the sensory panel). The jars were hermetically closed with a metal lid. The jars were then subjected to three pasteurization processes selected based on the Food and Drug Administration’s recommendations and previous reports [20]: 50 °C for 45 min (P1), 65 °C for 30 min (P2), and 70 °C for 15 min. Three jars from each pasteurization process were taken after pasteurization (Day 0) and after 20 and 35 days of storage at 4 °C for analytical determinations. The whole experiment was replicated three times.

### 2.5. Total Soluble Solids, pH, Titratable Acidity and Vitamin C Measures

Total soluble solids were measured in duplicate in each sample of citrus segments and syrup using a digital refractometer (Atago PR-101, Atago Co., Ltd., Tokyo, Japan) and the results were expressed as °Brix. Titratable acidity and pH were also measured in duplicate for each sample of segments and syrup by titration of 1 mL of juice (diluted in 25 mL of distilled water) up to pH 8.1 with an automatic titration system (785 DMP Titrino, Metrohm, Herisau, Switzerland). The results were expressed as g citric acid equivalent per 100 g or 100 mL of segments or juice, respectively. The vitamin C content in the segments and syrup was measured by a redox titration reaction with iodine following the method of Ciancaglini et al. [25], and results were expressed as mg 100 g^−1^ fresh weight or mg 100 mL^−1^ of juice, respectively. 

### 2.6. Microbiological and Sensory Analysis of the New Ready-to-Eat Product

Microbiological analysis was performed according to Sanchez-Bel et al. [12] and ISO-2001 [26]. Briefly, 10 g of segments or 10 mL of syrup were homogenized into a sterile stomacher bag containing 90 mL of 0.1% peptone (Merk, Darmstadt, Spain) for 90 s. Appropriate dilutions were made and mesophilic aerobic bacteria were measured after incubation on Plate Count Agar (PCA, Oxoid, Spain) at 35 ± 2 °C for 48 h. Psychrophilic aerobic bacteria were counted after incubation on the same PCA at 4 ± 1 °C for 5–15 days. Finally, yeast and moulds were counted after incubation on Rose-Bengal Chloranphenicol Agar plates (Oxoid, Spain) at 25 ± 2 °C for 5 days. Three replicate samples were assayed and the results are expressed as log colony forming units per gram (log CFU g^−1^). 

Sensory analysis was carried out by a panel of 10 semi-trained adults, as described previously in Section 2.3. Each panellist was served with one segment from each sample to evaluate colour, odour, acidity, sweetness, aroma, texture and overall impression on a ranked hedonic scale from 1 (not like at all) to 9 (like very much), according to Sanchez-Bel et al. [12]. For comparison with the segments of the new ready-to-eat product, the panellists were also served with one manually peeled segment of fresh fruit for which the maximum scores were given. 

### 2.7. Statistical Analysis

The results are presented as mean ± SD of three independent experiments or replicates. The data were subjected to analysis of variance (ANOVA) and the Tukey’s test was used for mean comparisons to examine if differences were significant at *p* < 0.05. All analyses were performed with SPSS software package version 11.0 for Windows.

## 3. Results and Discussion

### 3.1. Effect of Incubation Time on Quality Scores of Citrus Peeled Segments

In the present experiment, Peelzym II was used to obtain fruit segments from different fruit species and cultivars, which has been demonstrated to have higher peeling activity for citrus fruit compared with other enzymatic solutions [14]. This enzymatic peeling solution was applied in three pulses of two min under vacuum pressure and different subsequent periods of incubation were assayed because incubation times have a great impact on the quality of the obtained segments depending on the citrus fruit species and cultivar [12,13,14,27]. The percentage of not-attacked albedo was 0 for both clementine cultivars, which means that the albedo tissue from the whole fruit was totally removed by the peeling treatments, independently of the incubation time applied. For ‘Navel’ orange, more than 20 min of incubation time was needed for total albedo total removal, while for ‘Fino’ lemon, 30 min was needed and more than 30 min was necessary for ‘Star Ruby’ grapefruit (Table 1). The best results in terms of percentage of viable segments and global segment acceptation were obtained for ‘Hernandina’ clementine, with values close to 100% for both parameters and without significant differences among incubation times (Table 1). In addition, the maximum scores (5) were given for easiness of skin removal and easiness of segment separation, independently of the incubation time, while segment firmness decreased significantly (*p* < 0.05) with increased incubation time. Thus, for obtaining good quality segments of ‘Hernandina’ clementine by enzymatic peeling in the assayed experimental conditions, an incubation time of 10 min could be sufficient. High percentages of viable segments and global acceptance were also obtained for ‘Orogrande’ clementine, although 20 min of incubation time was needed to reach the highest scores for easiness of skin removing and segment separation. However, with this incubation time, segment firmness decreased significantly (*p* < 0.05) with respect to the 10 min treatment (Table 1). For ‘Navel’ orange, the scores for all the measured parameters (except for non-attacked albedo) increased significantly (*p* < 0.05) with the incubation time, and the highest values were reached with 30 min of treatment (Table 1). These results could be due to the presence of a navel in the fruit apical end of this orange cultivar which increases the time needed for the enzymatic solution to diffuse into the internal fruit tissues.

For ‘Fino’ lemon, an incubation time of 30 min was also needed to obtain the highest scores for all the assessed sensory parameters. In addition, it is worth noting that a high percentage of non-attacked albedo (≈25%) was found with 10 min of incubation time, although it decreased with increasing incubation time, and the entire fruit albedo was attacked by the enzymatic solution with 30 min of incubation time (Table 1). These results are attributed to the elongated shape of this lemon cultivar [28], which made it difficult to achieve homogeneous skin perforation when the fruits were rolled over the spiked wood plate, mainly at the two terminal ends of fruit. In contrast, the percentage of non-attacked albedo in ‘Star Ruby’ grapefruit ranged from 14 to 25%, without a clear effect of incubation time. In addition, none of the assayed incubation times led to reaching the highest scores for easiness of skin removal or segment separation as compared with other citrus fruit species or cultivars. Moreover, the percentages of viable segments and global acceptance decreased with incubation time, with values for the last parameter of ≈75, 78 and 65% for 10, 20 and 30 min, respectively (Table 1). Thus, to obtain segments with high-quality traits from this citrus cultivar, longer vacuum pulses or a greater number of them would be needed, but not an incubation time increase, since a significant softening of the segment was observed from 10 to 30 min of incubation in the present experimental conditions. The difficulty of obtaining enzymatically peeled segments from this citrus species could be attributed to the fact that it has a tough peel that is closely adhered to the inner fruit segments [29], which would limit the proper diffusion of the peeling solution.

**Figure 1:**
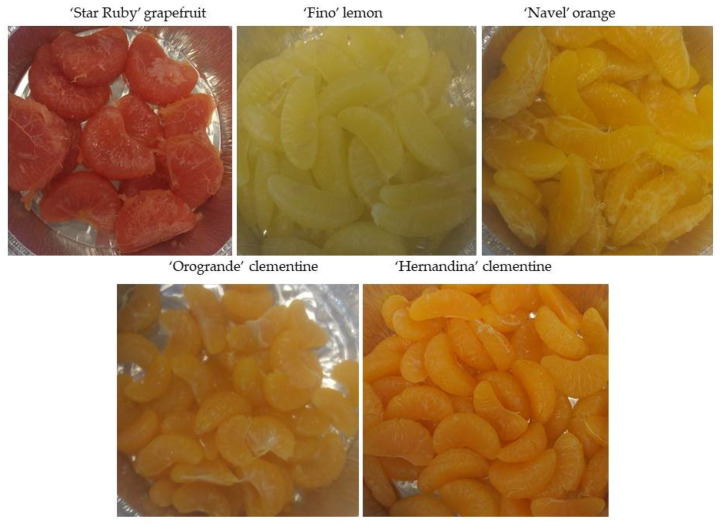
Photographs showing the visual appearance of the citrus segments obtained by enzymatic peeling with the best incubation time for each fruit: 10 min for ‘Star Ruby’ grape fruit and ‘Hernandina’ clementine, 20 min for ‘Orogrande’ clementine and 30 min for ‘Fino’ lemon and ‘Navel’ orange.

Considering these results and the visual appearance of the segments obtained from the different citrus species and cultivars (Figure 1), ‘Hernandina’ clementine was chosen to make a new ready-to-eat product based on citrus segments (obtained by enzymatic peeling with Peelzym II solution, applied at 1 g L^−1^ by three vacuum pulses of two min and a subsequent incubation time of 10 min) in a light syrup. 

### 3.2. Selection of Syrup to Elaborate the New Ready-to-Eat Clementine Segment Product

**Figure 2:**
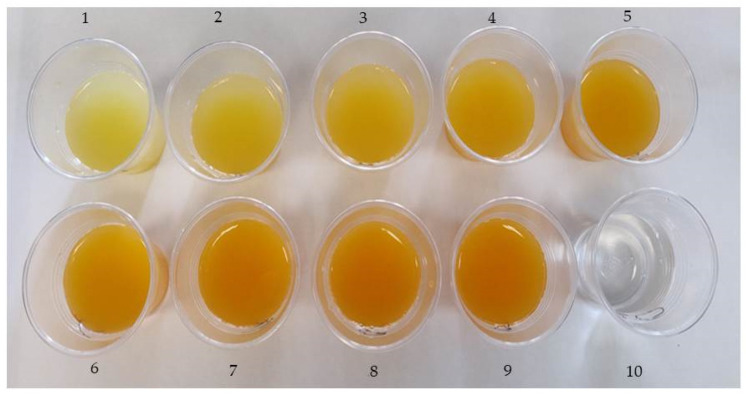
Photographs showing the visual appearance of the syrups made with different amount of ‘Fino’ lemon (FLJ) and ‘Hernandina’ clementine (HCJ) juices. 1: 90% FLJ/10% HCJ, 2: 80% FLJ/20% HCJ, 3: 70% FLJ/30% HCJ, 4: 60% FLJ/40% HCJ, 5: 50% FLJ/50% HCJ, 6: 40% FLJ/60% HCJ, 7: 30% FLJ/70% HCJ, 8: 20% FLJ/80% HCJ, 9: 10% FLJ/90% HCJ and 10: tap water with sugar cane up to 14 °Brix (control).

Syrup serves as an ideal preservation method for many ready-to-eat fruits and vegetables. According to the Spanish Real Decreto 2420/1978 (modified by Real Decreto 176/2013) [24] which aims to regulate the elaboration of canned vegetables, fruits in syrups are defined as products obtained from whole fruits, halves, segments, strips, cubes, slices or segments, to which a covering syrup has been added. In addition, these products are classified according to their sugar content in the final product, with those having 14 to 17 °Brix considered as light syrups. In the present experiment, different mixtures of ‘Fino’ lemon (FLJ) and ‘Hernandina’ clementine (HCJ) juices were made to form light syrups with the addition of cane sugar from organic farming up to final values of 14 °Brix. Tap water with the addition of sugar cane up to 14 °Brix was used as control. The visual aspect of the different syrups obtained with the juice mixtures after the addition of sugar cane is shown in Figure 2.

Values of °Brix for the FLJ–HCJ mixtures increased significantly (*p* < 0.05) as the lemon/clementine juice ratio decreased, ranging from ≈9 to ≈11 for 90% FLJ/10% HCJ and 10% FLJ/90% HCJ, respectively. The amount of sugar needed to be added to reach a 14 °Brix value was significantly reduced as the percentage of HCJ increased (Table 2). Significant (*p* < 0.05) increases in pH and decreases in TA were also observed in the syrups as the proportion of lemon juice decreased, with TA values of 5.26 ± 0.04 and 1.49 ± 0.11 g 100 mL^−1^ for syrups containing 90 and 10% of FLJ, respectively (Table 2).

A sensory analysis was performed with the obtained syrups to evaluate colour, sourness, sweetness, aroma, overall impression and purchase intention. Panellists gave higher scores for colour to the syrups with a higher content of HCJ (Table 3). This could be due to the fact that these mixtures have a similar colour to commercial orange juices, while the mixtures with a higher percentage of lemon juice with respect to clementine juice presented less attractive colours. Scores for sourness decreased as did the content of FLJ in the syrups, as expected. However, sweetness scores increased significantly from 1.50 ± 0.18 for syrup composed of 90% FLJ and 10%HCJ to 7.63 ± 0.32 for syrup made with 10% FLJ and 90% HCJ (Table 3), in spite of the fact that all the syrups had similar °Brix levels, that is to say, a similar sugar concentration. This could be attributed to the fact that the human sensory appreciation for sweetness and flavour depends on the TA/TSS ratio more than the levels of TSS itself [30]. Thus, syrups with a relatively high FLJ were more appreciated by the sensory panel that the lower sourness syrups. With respect to aroma scores, values ranged from 3.88 to 6.55, with the highest values being found for syrup samples containing 40–60% proportions of FLJ or HCJ, in which the characteristic aromas of lemon and clementine were detected in a balanced way (Table 3). Finally, for overall impression and purchase intention, scores given by the panellists increased significantly (*p* < 0.05) as the percentage of HCJ in the syrups increased up to 60% HCJ (with 40% FLJ), but these scores then decreased in syrups with higher HCJ content (Table 3). Thus, the syrup made of 50%FLJ + 50%HCJ was selected for further investigation of the ready-to-eat product with ‘Hernandina’ clementine segments since it had a good overall acceptance with the judges, giving balanced scores for all the evaluated sensory parameters.

### 3.3. Effect of Pasteurization Processes on Syrups and Segments Quality Traits during Storage

**Figure 3:**
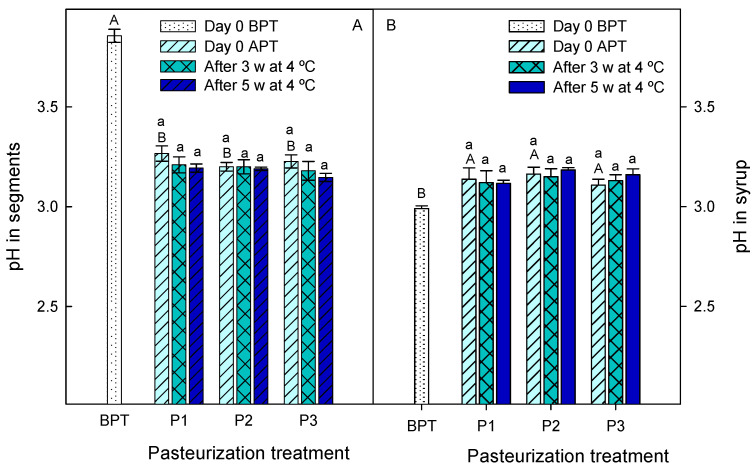
Values of pH in segments (**A**) and syrups (**B**) at day 0 before pasteurization treatments (BPT), at day 0 after pasteurization treatments (APT) and after 3 weeks (3 w) and 5 weeks (5 w) of storage at 4 °C. Pasteurization treatments: P1, 45 min at 50 °C; P2, 30 min at 65 °C and P3, 15 min at 70 °C. Data are the mean ± SD of three independent experiments. Different capital letters indicate significant differences (*p* < 0.05) between samples before and after pasteurization treatments, and different lower-case letters indicate significant differences during storage for each pasteurization treatment.

**Figure 4:**
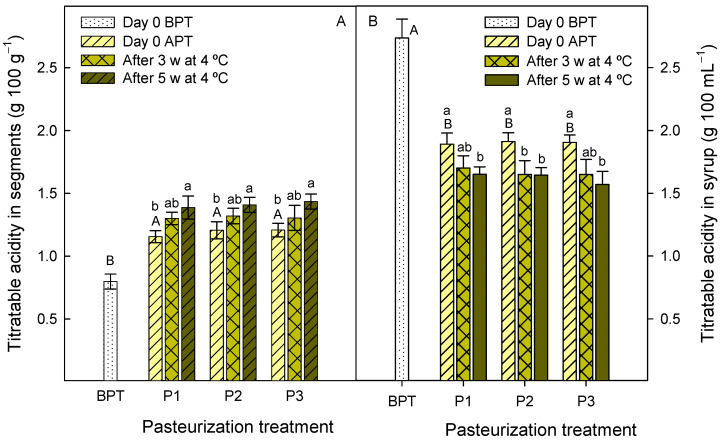
Titratable acidity in segments (**A**) and syrups (**B**) at day 0 before pasteurization treatments (BPT), at day 0 after pasteurization treatments (APT) and after 3 weeks (3 w) and 5 weeks (5 w) of storage at 4 °C. Pasteurization treatments: P1, 45 min at 50 °C; P2, 30 min at 65 °C and P3, 15 min at 70 °C. Data are the mean ± SD of three independent experiments. Different capital letters indicate significant differences (*p* < 0.05) between samples before and after pasteurization treatments, and different lower-case letters indicate significant differences during storage for each pasteurization treatment.

The ready-to-eat product composed of enzymatically peeled segments of ‘Hernadina’ clementine and the 50% FLJ + 50% HCJ syrup was subjected to three different pasteurization treatments and its quality parameters and microbial safety were evaluated during storage at 4 °C for 5 weeks. Concentrations of organic acids and sugars of citrus fruit and juices are usually used as physico-chemical quality indicators, although their values depend on several factors, such as cultivar, growing conditions, environmental factors and fruit maturation, among others [17,31]. The syrup and segment pH values were lower than 4.6 before and after the pasteurization treatments (Figure 3), which is the limiting threshold for bacterial growth [32]. Therefore, all the pasteurization treatments would be useful to ensure food safety of the new ready-to-eat product. The pH in the ‘Hernandine’ clementine segments was 3.85 ± 0.03 before the pasteurization process and decreased significantly to ≈3.2 after pasteurization without significant differences among pasteurization treatments. These pH values were maintained at similar levels (*p* > 0.05) during the storage at 4 °C (Figure 3A). For the syrup, the pH before pasteurization was 2.99 ± 0.01 and increased significantly (*p* < 0.05) after pasteurization, although no significant differences were observed among pasteurization treatments or storage times (Figure 3B). An opposite trend was observed for TA, that is to say, significant increases (*p* < 0.05) immediately after pasteurization were observed for segments (from 0.80 ± 0.06 to ≈0.31 g 100 g^−1^) and significant decreases (*p* < 0.05) for syrups (from 2.74 ± 0.15 to ≈1.70 g 100 mL^−1^), independently of the pasteurization treatment applied (Figure 4). In addition, significant changes (*p* < 0.05) in TA were also observed during storage, with increases of 12, 17 and 19% for P1, P2 and P3 segment pasteurization treatments, respectively, while for the syrups, there were 13, 14 and 18% decreases, respectively (Figure 4).

**Figure 5:**
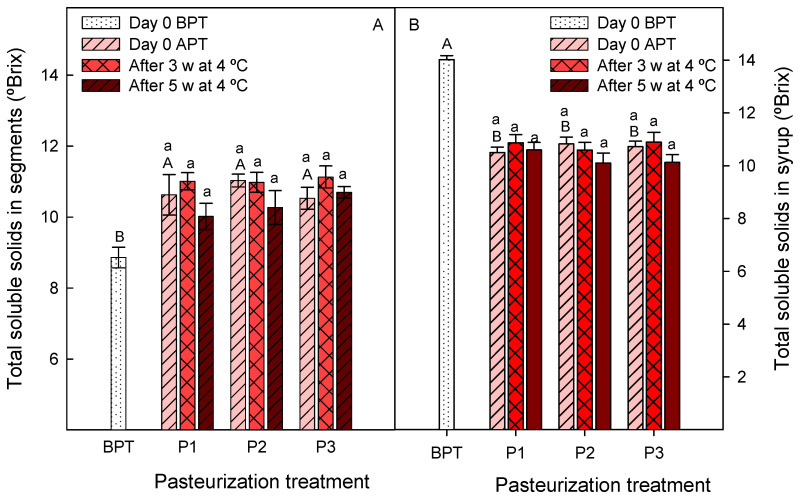
Total soluble solid concentrations in segments (**A**) and syrups (**B**) at day 0 before pasteurization treatments (BPT), at day 0 after pasteurization treatments (APT) and after 3 weeks (3 w) and 5 weeks (5 w) of storage at 4 °C. Pasteurization treatments: P1, 45 min at 50 °C; P2, 30 min at 65 °C and P3, 15 min at 70 °C. Data are the mean ± SD of three independent experiments. Different capital letters indicate significant differences (*p* < 0.05) between samples before and after pasteurization treatments, and different lower-case letters indicate significant differences during storage for each pasteurization treatment.

Significant changes (*p* < 0.05) were also observed in TSS after pasteurization treatments, both in segments and in syrups, from 8.9 to 11 °Brix in segments (Figure 5A) and from 14 to 10.6–10.9 °Brix in syrup (Figure 5B). However, the values of TSS were maintained at similar levels (*p* > 0.05) during storage in both segments and syrup (Figure 5A,B). The results show that the pasteurization treatments led to a diffusion of organic acids and sugars from syrup to the clementine segments, leading to increases in TSS and TA in the segments and decreases in the syrup, these changes being higher immediately after pasteurization than over the whole storage time for five weeks. In addition, a decrease in TA in juice during storage could have occurred per se since it is a general trend observed for citrus juices [33,34].

**Figure 6:**
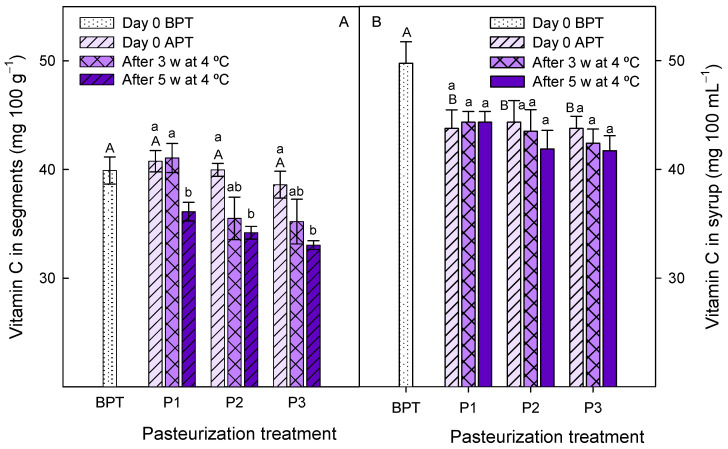
Vitamin C concentrations in segments (**A**) and syrups (**B**) at day 0 before pasteurization treatments (BPT), at day 0 after pasteurization treatments (APT) and after 3 weeks (3 w) and 5 weeks (5 w) of storage at 4 °C. Pasteurization treatments: P1, 45 min at 50 °C; P2, 30 min at 65 °C and P3, 15 min at 70 °C. Data are the mean ± SD of three independent experiments. Different capital letters indicate significant differences (*p* < 0.05) between samples before and after pasteurization treatments, and different lower-case letters indicate significant differences during storage for each pasteurization treatment.

The vitamin C concentration in fresh segments was 39.90 ± 1.25 mg 100 g^−1^, and similar values were observed after the pasteurization processes. However, decreases in vitamin C content were observed during storage in all segment samples, which were significant (*p* < 0.05) after 35 days of cold storage compared with data immediately after pasteurization (Figure 6A). In contrast, the vitamin C concentration in syrup was 49.76 ± 1.98 mg 100 mL^−1^ before pasteurization and a significant (*p* < 0.05) decrease, ca. 12%, was observed after pasteurization, while no changes occurred during the whole storage period (Figure 6B). Vitamin C, which is found at high concentrations in citrus fruit, is an essential vitamin for humans with important effects on improving the immune system, leading to reduced risks of several diseases, such as heart disease, infectious illnesses and several kinds of cancer, among others [3,35]. Apart from vitamin C, citrus fruit contains a wide range of other antioxidant bioactive compounds, mainly carotenoids and phenolics, which differ qualitatively and quantitatively depending on the fruit species and cultivars, and collectively are responsible for health beneficial properties [3,36,37]. In ‘Fino’ lemon fruits, increases in total phenolics content during cold storage have been reported, while decreases were found in ascorbic acid concentration [38,39]. Accordingly, 30% loses in ascorbic acid have been reported in ‘Satsuma’ mandarin fruit after 35 days of storage at 4 °C [40], and 25% loses were reported in manually obtained segments after 9 days of cold storage in low density polyethylene bags [41]. On the other hand, the pasteurization process impacts quality and nutritional components as well as antioxidant compounds of vegetable products, with general reductions depending on the temperature used and time ranges [20]. For instance, thermal pasteurization (at 70 °C for 30 s) of orange juice led to a 25–30% decrease in total phenolic, flavonoid, anthocyanin and carotenoid concentrations immediately after pasteurization and during subsequent cold storage for 35 days [42]. In ‘Nagpur’ mandarins, juice pasteurization at 65 or 75 °C for 10–35 min resulted in a 30% reduction in vitamin C content [34]. Accordingly, Cheng et al. [33] reported that after thermal pasteurization of mandarin juice at 90 °C for 30 s ca., 12% losses of vitamin C content occurred, due to oxidative reactions, as well as 40 and 60% losses for carotenoids and phenolics, respectively. Moreover, pasteurization of orange juice for 20 s at 90 °C, which is the normal commercial practice, led to 50% vitamin C losses after 5 weeks of storage [43]. Degradation of vitamin C and phenolics, including anthocyanins, is a common occurrence during juice pasteurization and has been described as a first-order model, during heating at 80, 90 or 100 °C for 6 to 22 min, in oranges, lemons, grapefruits and strawberries [44,45,46]. However, it is worth noting that in the present experiment, losses of vitamin C in the syrup and segments were lower than losses reported in previous papers, showing that the pasteurization process selected for this new ready-to-eat product was optimum for preserving this antioxidant and valuable compounds.

### 3.4. Sensory and Microbiological Quality

‘Hernandina’ clementine, a hybrid between tangerine (*Citrus reticulata* Blanco) and sweet orange (*Citrus sinensis* (L.) Osbeck), is a citrus fruit very appreciated by consumers due to its high-quality traits, such as taste, aroma and nutritive and antioxidant compounds [47]. Pasteurization treatments significantly affected (*p* < 0.05) scores for sensory parameters, with higher values for P1 compared with P2 and P3, except for odour and sweetness (Table 4). In the P1 treatments samples, significant (*p* < 0.05) increases occurred in odour scores after 21 days of storage, while no changes were observed in samples for P2 and P3. Scores given by panellists for segment colour were also significantly higher for the P1 treatment than for the P2 and P3 treatments, at day 0 and after 21 and 35 days of storage, although no significant changes in colour were observed during storage (Table 4). Acidity, sweetness and aroma sensory parameters were evaluated only at day 0 for safety reasons; the judges needed to eat the segments to evaluate them, and after 21 or 35 days of storage, prior microbial analysis would be necessary to ensure the safety of the samples. Nevertheless, these parameters obtained significantly (*p* < 0.05) higher scores for P1 pasteurization treatments than for P2 and P3. The scores for texture were significantly (*p* < 0.05) affected by pasteurization treatments, with values of 6.50 ± 0.23 for P1, 4.50 ± 0.37 for P2 and 3.50 ± 0.20 for P3 immediately after the pasteurization process (Table 4). Increases in scores for texture were found during storage, although they were significant (*p* < 0.05) only for samples of the P1 treatment. Finally, scores for overall impression were significantly (*p* < 0.05) higher for samples of the P1 treatment, compared with P2 and P3, at day 0 after pasteurization as well as after 21 and 35 days of storage (Table 4). During storage, the scores for overall impression of the P1 samples increased significantly (*p* < 0.05), reaching values of 8.02 ± 0.20 after 35 days of storage, while the increases were not significant for the P2 and P3 treatment samples (Table 4).

The new ready-to-eat product was tested for microbiological quality by measuring mesophilic aerobic bacteria, psychrophilic aerobic bacteria and yeast and mould in segments at day 0 before and after the peeling process and in syrups before and after the pasteurization treatments, as well as in segments and syrups after 3 and 5 weeks of storage at 4 °C. The results showed that microbial counts for mesophilic aerobic bacteria and for mould and yeast ranged from 2 to 3.7 log CFU in both segments and syrup at day 0 before the pasteurization treatments, while for psychrophilic aerobic bacteria, the counts were <1 log UFC (Table 5). However, no microbial contamination was detected, either in the segments or in syrups during the whole storage time since the microbial counts were null for mesophilic and psychrophilic bacteria, as well as for yeast and mould (Table 5). Thus, P1 pasteurization treatment (45 min at 50 °C) could be sufficient to ensure the microbiological safety of both syrup and segments in this ready-to-eat product [20]. Moreover, this new product conserved its safety and high nutritive and organoleptic properties until the end of storage and could be a good alternative for satisfying consumers’ demands for convenient and healthy foods from sourced from local and organic produce [5,6,48]. In this context, it is worth noting that the citrus fruits and sugar cane used in the present experiments were obtained from commercial companies following the rules of Commission Implementing Regulation (EU) 2021/279 of 22 February 2021, regarding organic farming and labelling of organic products [49]. (https://www.caecv.com/normativa/ (accessed on 28 August 2023)).

## 4. Conclusions

The highest segment quality obtained for enzymatic peeling was observed for ‘Hernandina’ clementine, which was chosen to make a new ready-to-eat product based on these segments packaged in glass jars with a light syrup made of 50%FLJ and 50%HCJ and added sugar cane until 14 °Brix. This syrup was selected based on the sensory scores given by panellists compared with syrups made with 10–90% combinations of fruit both juices. Thereafter, different pasteurization treatments were assayed, and the results showed that a pasteurization treatment at 50 °C for 45 min was sufficient to prevent mesophilic and psychrophilic aerobic bacteria or yeast and mould contamination and to maintain sensory properties until 35 days of storage at 4 °C. In addition, only a 10% reduction in vitamin C was observed when comparing fresh citrus fruit segments or syrup with products at the end of the storage period. Thus, this new ready-to-eat product is safe and conserves high levels of sensory properties and bioactive compounds as well as health benefits after cold storage for at least seven weeks and would have potential application in the agro-food industry. However, further research should be performed to determine if the storage period could be extended for longer periods. Future work could also investigate ready-to-eat products based on other citrus species and cultivars, as well as fruit segments and syrups with different colours and aroma for application in the agro-food industry.

## Figures and Tables

**Table 1:** Effects of incubation time on easiness of segment separation, easiness of skin removal and segment firmness (assigned values from 0 to 5), and percentages of non-attacked albedo, viable segments and global acceptance of the segments obtained by enzymatic peeling of different citrus fruit cultivars.

Citrus Cultivar	Incubation Time (min)	Non-Attacked Albedo (%)	Easiness of Skin Removing	Easiness of Segment Separation	Segment Firmness	Viable Segments (%)	Global Acceptance (%)
‘Star Ruby’ grapefruit	10	16.01 ± 1.74 ^b^	2.61 ± 0.29 ^c^	2.01 ± 0.63 ^b^	4.02 ± 0.23 ^a^	80.01 ± 3.21 ^a^	75.01 ± 3.16 ^a^
	20	25.25 ± 1.32 ^a^	3.09 ± 0.33 ^b^	2.29 ± 0.40 ^b^	3.64 ± 0.13 ^b^	84.27 ± 4.02 ^a^	78.17 ± 3.16 ^a^
	30	14.18 ± 1.63 ^b^	4.22 ± 0.20 ^a^	4.41 ± 0.49 ^a^	3.09 ± 0.29 ^c^	73.04 ± 3.61 ^b^	65.02 ± 2.78 ^b^
‘Fino’ lemon	10	24.08 ± 2.53 ^a^	3.61 ± 0.49 ^c^	3.42 ± 0.49 ^c^	3.82 ± 0.40 ^c^	82.04 ± 2.48 ^b^	83.24 ± 2.48 ^b^
	20	7.37 ± 0.78 ^b^	4.42 ± 0.80 ^b^	4.27 ± 0.98 ^b^	4.41 ± 0.49 ^b^	86.25 ± 2.40 ^b^	84.38 ± 4.20 ^b^
	30	0 ^c^	5 ± 0 ^a^	5 ± 0 ^a^	5 ± 0 ^a^	98.01 ± 4.02 ^a^	98.09 ± 4.21 ^a^
‘Navel’ orange	10	3.02 ± 0.45 ^a^	4.40 ± 0.19 ^b^	4.41 ± 0.29 ^b^	3.44 ± 0.29 ^c^	83.27 ± 3.04 ^c^	85.01 ± 2.47 ^c^
	20	0 ^b^	4.30 ± 0.24 ^b^	4.77 ± 0.40 ^b^	4.01 ± 0 ^b^	90.05 ± 3.16 ^b^	90.87 ± 2.05 ^b^
	30	0 ^b^	5 ± 0 ^a^	5 ± 0 ^a^	5 ± 0 ^a^	100 ± 0 ^a^	100 ± 0 ^a^
‘Orogrande’ clementine	10	0	4.41 ± 0.19 ^b^	2.81 ± 2.31 ^b^	5 ± 0 ^a^	100 ± 0 ^a^	96.34 ± 2.21 ^a^
	20	0	5 ± 0 ^a^	5 ± 0 ^a^	3.80 ± 0.17 ^b^	93.14 ± 3.12 ^b^	95.31 ± 3.12 ^a^
	30	0	5 ± 0 ^a^	5 ± 0 ^a^	3.01 ± 0.14 ^c^	73.02 ± 3.78 ^c^	73.06 ± 3.16 ^b^
‘Hernandina’ clementine	10	0	5 ± 0 ^a^	5 ± 0 ^a^	5 ± 0 ^a^	100 ± 0 ^a^	98.21 ± 1.74 ^a^
	20	0	5 ± 0 ^a^	5 ± 0 ^a^	4.62 ± 0.25 ^b^	100 ± 0 ^a^	97.36 ± 1.45 ^a^
	30	0	5 ± 0 ^a^	5 ± 0 ^a^	4.03 ± 0.17 ^c^	97.07 ± 1.24 ^a^	96.24 ± 2.74 ^a^

Data are the mean ± SD of three independent experiments. Different letters within each column indicate significant differences among incubation time treatments for each citrus cultivar.

**Table 2:** Percentages of ‘Fino’ lemon juice (FLJ) and ‘Hernandina’ clementine juice (HCJ) used to make the syrup, initial total soluble solids (TSS, °Brix) of the juice mixture, amount of sugar added (g 100 mL^−1^) to reach a final value of 14 °Brix in the syrup, and pH and titratable acidity (TA g 100 mL^−1^) in the obtained syrup.

FLJ and HCJ Percentages	Initial TSS (°Brix)	Sugar Added (g 100 mL^−1^)	pH of the Syrups	TA (g 100 mL^−1^) of the Syrups
90% FLJ/10% HCJ	9.12 ± 0.12 ^f^	5.71 ± 0.31 ^a^	2.24 ± 0.35 ^d^	5.26 ± 0.04 ^a^
80% FLJ/20% HCJ	9.43 ± 0.19 ^ef^	5.12 ± 0.23 ^ab^	2.71 ± 0.28 ^c^	4.85 ± 0.10 ^b^
70% FLJ/30% HCJ	9.74 ± 0.23 ^de^	4.75 ± 0.21 ^bc^	2.75 ± 0.19 ^c^	4.37 ± 0.06 ^c^
60% FLJ/40% HCJ	9.96 ± 0.27 ^cde^	4.51 ± 0.17 ^ce^	2.84 ± 0.32 ^bc^	4.01 ± 0.08 ^d^
50% FLJ/50% HCJ	10.31 ± 0.29 ^bcd^	4.36 ± 0.15 ^ce^	2.88 ± 0.23 ^bc^	3.30 ± 0.13 ^e^
40% FLJ/60% HCJ	10.53 ± 0.33 ^bc^	4.21 ± 0.11 ^ef^	2.93 ± 0.15 ^ab^	2.92 ± 0.08 ^f^
30% FLJ/70% HCJ	10.71 ± 0.28 ^ab^	4.09 ± 0.09 ^efg^	2.99 ± 0.16 ^ab^	2.44 ± 0.09 ^g^
20% FLJ/80% HCJ	10.87 ± 0.29 ^ab^	3.79 ± 0.11 ^fgh^	3.10 ± 0.10 ^a^	1.91 ± 0.08 ^h^
10% FLJ/90% HCJ	11.12 ± 0.27 ^a^	3.61 ± 0.12 ^g^	3.30 ± 0.25 ^a^	1.49 ± 0.11 ^i^
Control ^b^	0	18.5	-	-

Data are the mean ± SD of three independent experiments. Different letters within each column for each measured parameter indicate significant differences among syrups.

**Table 3:** Sensory analysis scores (from 0, do not like at all, to 10, like very much) for the syrups made with different percentages of ‘Fino’ lemon juice (FLJ) and ‘Hernandina’ clementine juice (HCJ). Tap water with added sugar up to 14 °Brix was used as a control.

Syrups (%FLJ/%HCJ)	Colour	Sourness	Sweetness	Aroma	Overall Impression	Purchase Intention
90% FLJ/10%HCJ	4.88 ± 0.28 ^f^	9.75 ± 0.20 ^a^	1.50 ± 0.18 ^h^	5.00 ± 0.40 ^b^	3.75 ± 0.30 ^d^	3.75 ± 0.32 ^c^
80% FLJ/20% HCJ	5.00 ± 0.19 ^f^	9.38 ± 0.34 ^ab^	1.63 ± 0.13 ^h^	5.13 ± 0.56 ^b^	3.38 ± 0.20 ^d^	3.63 ± 0.27 ^c^
70% FLJ/30% HCJ	5.63 ± 0.23 ^e^	8.62 ± 0.38 ^b^	2.75 ± 0.31 ^g^	4.90 ± 0.42 ^b^	4.63 ± 0.23 ^c^	3.88 ± 0.22 ^c^
60% FLJ/40% HCJ	5.63 ± 0.27 ^e^	7.38 ± 0.24 ^c^	3.13 ± 0.27 ^g^	5.63 ± 0.32 ^ab^	6.35 ± 0.20 ^a^	5.13 ± 0.26 ^b^
50% FLJ/50% HCJ	7.00 ± 0.16 ^d^	6.63 ± 0.23 ^d^	4.13 ± 0.14 ^f^	6.55 ± 0.43 ^a^	6.53 ± 0.15 ^a^	6.25 ± 0.15 ^a^
40% FLJ/60% HCJ	7.50 ± 0.22 ^c^	6.38 ± 0.19 ^e^	4.88 ± 0.28 ^e^	5.88 ± 0.52 ^ab^	6.88 ± 0.22 ^a^	6.38 ± 0.15 ^a^
30% FLJ/70% HCJ	8.00 ± 0.16 ^b^	5.25 ± 0.29 ^f^	6.00 ± 0.17 ^d^	5.13 ± 0.37 ^b^	6.63 ± 0.29 ^a^	5.13 ± 0.04 ^b^
20% FLJ/80% HCJ	8.88 ± 0.29 ^a^	4.13 ± 0.16 ^g^	6.63 ± 0.22 ^c^	4.38 ± 0.24 ^bc^	5.38 ± 0.32 ^b^	5.38 ± 0.11 ^b^
10% FLJ/90% HCJ	9.38 ± 0.21 ^a^	3.13 ± 0.22 ^h^	7.63 ± 0.32 ^b^	3.88 ± 0.26 ^c^	5.50 ± 0.28 ^b^	5.63 ± 0.26 ^b^
Control	5.00 ± 0.25 ^f^	0 ^i^	9.50 ± 0.27 ^a^	1.13 ± 0.11 ^d^	2.63 ± 0.11 ^e^	2.63 ± 0.08 ^d^

Data are the mean ± SD of three independent experiments. Different letters within each column for each sensory parameter indicate significant differences among syrups.

**Table 4:** Sensory scores for ‘Hernandina’ clementine segments of the ready-to-eat product as affected by pasteurization treatment (P1, 45 min at 50 °C; P2, 30 min at 65 °C and P3, 15 min at 70 °C) and storage time at 4 °C.

Storage Time and Pasteurization Treatment	Odour	Colour	Acidity	Sweetness	Aroma	Texture	Overall Impression
Day 0 P1	6.50 ± 0.32 ^aB^	8.00 ± 0.23 ^aA^	7.25 ± 0.49 ^a^	5.31 ± 0.24 ^a^	5.50 ± 0.17 ^a^	6.50 ± 0.23 ^aB^	6.25 ± 0.26 ^aB^
Day 0 P2	6.10 ± 0.22 ^aA^	7.25 ± 0.28 ^bA^	6.75 ± 0.36 ^a^	5.62 ± 0.62 ^a^	4.75 ± 0.36 ^b^	4.50 ± 0.37 ^bA^	5.25 ± 0.36 ^bA^
Day 0 P3	6.25 ± 0.86 ^aA^	7.00 ± 0.41 ^bA^	6.65 ± 0.44 ^a^	5.28 ± 0.47 ^a^	4.50 ± 0.50 ^b^	3.50 ± 0.20 ^cA^	5.03 ± 0.24 ^bA^
21 Days 4 °C P1	7.25 ± 0.23 ^aA^	8.25 ± 0.43 ^aA^	-	-	-	7.00 ± 0.27 ^aAB^	7.75 ± 0.34 ^aA^
21 Days 4 °C P2	6.75 ± 0.37 ^abA^	7.00 ± 0.4 ^bA^	-	-	-	4.75 ± 0.28 ^bA^	5.75 ± 0.28 ^bA^
21 Days 4 °C P3	6.50 ± 0.32 ^bA^	6.75 ± 0.49 ^bA^	-	-	-	4.00 ± 0.24 ^cA^	5.50 ± 0.17 ^bA^
35 Days 4 °C P1	7.50 ± 0.3 ^aA^	8.50 ± 0.37 ^aA^	-	-	-	7.30 ± 0.35 ^aA^	8.02 ± 0.20 ^aA^
35 Days 4 °C P2	6.25 ± 0.28 ^bA^	6.75 ± 0.36 ^bA^	-	-	-	4.75 ± 0.28 ^bA^	5.70 ± 0.25 ^bA^
35 Days 4 °C P3	6.75 ± 0.25 ^bA^	6.50 ± 0.55 ^bA^	-	-	-	3.75 ± 0.36 ^cA^	5.51 ± 0.19 ^bA^

Data are the mean ± SD of three replicates. Different lower-case letters indicate significant differences among pasteurization treatments at *p* < 0.05 for each sampling date and different capital letters indicate significant differences at *p* < 0.05 for each treatment along storage time.

**Table 5:** Values of mesophilic aerobic bacteria, psychrophilic aerobic bacteria, and yeast and moulds (log UFC g^−1^) in segments at day 0 before the peeling process (BPP), after peeling (APP) and after pasteurization treatments (BPT). Values in syrups before and after pasteurization treatments (BPT and APT), and microbial counts in segments and syrups after 3 (3 w) or 5 weeks (5 w) of storage at 4 °C. Pasteurization treatments: P1, 45 min at 50 °C; P2, 30 min at 65 °C and P3, 15 min at 70 °C. Data are the mean ± SD of three independent experiments.

	Pasteurization Treatments	Mesophilic Aerobic (log CFU g^−1^)	Pscycrophylic Aerobic (log CFU g^−1^)	Mould and Yeast (log CFU g^−1^)
Day 0 in segments BPP	-	3.71 ± 0.20 ^a^	<1	3.03 ± 0.21 ^a^
Day 0 in segments APP	-	3.23 ± 0.21 ^b^	<1	2.10 ± 0.20 ^b^
Day 0 in syrup BPT		2.69 ± 0.17	<1	2.02 ± 0.15 ^b^
Day 0 APT and after 3 or 5 w at 4 °C in segments and syrup	P1	0 ^c^		0 ^c^
	P2	0 ^c^		0 ^c^
	P3	0 ^c^		0 ^c^

Data are the mean ± SD of three replicates. Different letters within a column indicate significant differences (*p* < 0.05) between samples before and after peeling or pasteurization treatments.

## Data Availability

The data presented in this study are available upon request from the corresponding author.
